# Estimation specific food allergy in children from Sarajevo

**DOI:** 10.1186/2045-7022-1-S1-P80

**Published:** 2011-08-12

**Authors:** Adnan Bajraktarevic, Semira Penava, Begler Begovic, Goran Todosijevic, Amina Selimovic, Zijo Begic, Haris Niksic, Teodora Frankic, Aida Djulepa Djurdjevic, Aida Resic Secerbegovic, Lutvo Sporisevic, Branka Djukic

**Affiliations:** 1 Public Health Institution of Canton Sarajevo, Pediatrics Department, Sarajevo, Bosnia and Herzegovina; 2 Clinical Medical Center Sarajevo, Clinical Pharmacology, Sarajevo, Bosnia and Herzegovina; 3 Clinical Medical Center Sarajevo, Emergency Department, Sarajevo, Bosnia and Herzegovina; 4 Pediatrics Clinic Sarajevo, Department for allergology and pulmonology, Sarajevo, Bosnia and Herzegovina; 5 Pediatrics Clinic Sarajevo, Cardiology Department, Sarajevo, Bosnia and Herzegovina; 6 Pharmacy Faculty Sarajevo, Clinical Pharmacology, Sarajevo, Bosnia and Herzegovina; 7 General Hospital Sarajevo, Emergency Department, Sarajevo, Bosnia and Herzegovina; 8 Dermatologic Clinic Sarajevo, Allergology Department, Sarajevo, Bosnia and Herzegovina; 9 First Medical Aid New Sarajevo, Pediatrics Department, Sarajevo, Bosnia and Herzegovina

## Introduction

Food allergy most often begins in the first 1 to 2 years of life with the process of sensitization, by which the immune system responds to specific food proteins. Symptoms of a food allergy reaction commonly involve localized hives and worsening eczema, with moderate-to-severe atopic dermatitis a frequent comorbid condition of food allergy. Acute urticaria is much more likely to be caused by food allergy than is chronic urticaria.

## Methods

A blood test measured childr immune system's response to particular foods by checking the amount of allergy-type antibodies in bloodstream, known as immunoglobulin E (IgE) antibodies. Allergy testing was otherwise conducted in more than 1000 children during period 2000-2010 in Sarajevo, Bosnia and Herzegovina on Department for Allergy Testing.

## Results

Overall, 19% of the children were reported to have an adverse food reaction, and the reactions were confirmed by challenge in 6%. In that study,industrial meat and canned meat as sausages had been outgrown 369 of 1000 allergic children (about 37%) , egg allergy had been in 261 of 1000 children patients (26%), compared with 61 of 1000 with milk allergy (6%), 35 of 1000 with strawberry allergy (3.5%), 25 of 1000 with peanut allergy (2.5%) and other food allergy as citrus fruits, milk allergy, soy allergy, other nuts allergy, fish, tomato,herbal allergy, wheat allergy and other allergy had been in 249 of 1000 cases (about 25%). The duration of the reactions overall was short, with approximately two thirds of the reactions resolving within six months of their onset.

## Conclusion

Six percent of Bosnian children have documented food allergy. Currently available diagnostic methods for food allergy, such as prick skin tests and serum food allergen-specific IgE levels, do not distinguish between children who will achieve food tolerance and those who will have persistent food allergy. The diagnostic approach to the child patient should parallel those used in diagnosis of other adverse reactions to foods.

## Key Words

Allergy, Food, Children, Therapy

